# C-kit signaling promotes human pre-implantation 3PN embryonic development and blastocyst formation

**DOI:** 10.1186/s12958-019-0521-8

**Published:** 2019-09-10

**Authors:** Jun Tan, Yang Zou, Zhi-Hui Huang, Zhi-Qin Zhang, Li-Ping Wu, Xing-Wu Wu, Xiao-Ju Wan, Cai-Lin Xin, Qiong-Fang Wu

**Affiliations:** 1 Reproductive Medicine Center, Jiangxi Provincial Maternal and Child Health Hospital, Nanchang, Jiangxi 330006 People’s Republic of China; 2 Key Laboratory of Women’s Reproductive Health of Jiangxi Province, Jiangxi Provincial Maternal and Child Health Hospital, Nanchang, Jiangxi 330006 People’s Republic of China

**Keywords:** C-kit, ETV5, Embryonic development, In vitro culture, IVF

## Abstract

**Background:**

Although in vitro culture system has been optimized in the past few decades, the problem of few or no high quality embryos has been still not completely solved. Accordingly, fully understanding the regulatory mechanism of pre-implantation embryonic development would be beneficial to further optimize the in vitro embryo culture system. Recent studies have found the expression of c-kit in mouse embryo and its promotion effects on mouse embryonic development. However, it is unclear the expression, the role and the related molecular regulatory mechanism of c-kit in human pre-implantation embryo development. Therefore, the present study is to determine whether c-kit is expressed in human pre-implantation embryos, and to investigate the possible regulatory mechanism of c-kit signaling in the process of embryonic development.

**Methods:**

The present study includes human immature oocytes and three pronucleus (3PN) embryos collected from 768 women (28–32 ages) undergoing IVF, and normal 2PN embryos collected from ICR mice. Samples were distributed randomly into three different experimental groups: SCF group: G-1™ (medium for culture of embryos from the pro-nucleate stage to day 3) or G-2™ (medium for culture of embryos from day3 to blastocyst stage) + HSA (Human serum album) solution + rhSCF; SCF + imanitib (c-kit inhibitor) group: G-1™ or G-2™ + HSA solution + rhSCF + imanitib; SCF + U0126 (MEK/ERK inhibitor) group: G-1™ or G-2™ + HSA solution + rhSCF + U0126; Control group: G-1™ or G-2™ + HSA solution + PBS; The rate of good quality embryos at day 3, blastulation at day 6 and good quality blastulation at day 6 were analysis. RT-PCR, western blot and immunofluorescence staining were applied to detect the target genes and proteins in samples collected from human or mice, respectively.

**Results:**

c-kit was expressed ubiquitously in all human immature oocytes, 3PN embryos and 3PN blastocysts. In the experiment of human 3PN embryos, compared with other groups, SCF group showed obviously higher rate of good quality at day 3, better rate of blastocyst formation at day 6 and higher rate of good quality blastocyst formation at day 6. Furthermore, we observed a higher ETV5 expression in SCF group than that in other groups. Similar results were also found in animal experiment. Interestingly, we also found a higher phosphorylation level of MEK/ERK signal molecule in mice embryos from SCF group than those from other groups. Moreover, inhibition of MEK/ERK signaling would remarkably impeded the mice embryonic development, which might be due to the reduced ETV5 expression.

**Conclusions:**

The present study firstly revealed that c-kit signaling might promote the human pre-implantation embryonic development and blastocyst formation by up-regulating the expression of ETV5 via MEK/ERK pathway. Our findings provide a new idea for optimizing the in vitro embryo culture condition during ART program, which is beneficial to obtain high quality embryos for infertile patients.

## Background

In vitro fertilization-embryo transfer (IVF-ET) has become the main approach to solve the problem of infertility [1]. In the process of IVF-ET, it is important to generate high quality embryos as this is a major factor to obtain pregnancy and life births [2]. Therefore, further optimization of culture conditions can help to improve pregnancy rate.

It has been acknowledged that multiple types of receptors expressed on the surface of the embryo play a key role in embryonic early development [3, 4], for example, growth factor receptors (GFRs), cytokine receptors (CRs) and hormone receptors (HRs) [5–7]. Furthermore, animal experiments showed that co-cultured with agonists or ligands of receptor tyrosine kinases (RTKs), including epidermal growth factor (EGF), colony stimulating factor (CSF), insulin like growth factor-1 (IGF-1) and so on, could remarkably promote embryo development and improve embryo quality [8–12]. Whereas, inhibition of RTKs function would weaken embryo growth ability resulting in arrest of development and even embryo degeneration [13–17]. These results suggest that RTKs are associated with the early embryonic development [18–21].

C-kit, a member of the RTK family, can be activated by its ligand, stem cell factor (SCF). Activation of c-kit signaling plays an important role in the proliferation, differentiation, migration and apoptosis of many kinds of cells, such as melanocyte, mast cell, Cajal cell and hemopoietic stem cell [22, 23]. Previous studies have found the expression of c-kit receptor in oocytes from human and mouse, moreover, the signaling pathways downstream of c-kit play critical role in events like primordial follicle activation and follicular growth [24, 25]. Recently, Taniguchi et al. have detected the expression of c-kit receptor in mouse embryo [26]. Subsequently, in vitro co-cultured mouse embryos with SCF showed that activation of c-kit signaling significantly accelerated the embryonic development and supported the blastocyst formation [27]. So far it is unknown whether c-kit receptor is also expressed in human embryos and how it affects the embryonic development.

PEA3 transcription factors belong to the subfamily of E26 Transformation-Specific (ETS) transcription factors family. PEA3 subfamily includes three members, ETV1, ETV4 and ETV5 [28]. All the members are highly conserved at the N- and C-terminus in acidic domain, which constitutes the core of a transcription activation domain, and are capable of inducing activation of target genes [28]. Several studies have proved that PEA3 transcription factors correlated with multiple types of mouse organs development [29–31]. The role of ETV5 in male reproduction has been revealed by using etv5^−/−^ male mice [32]. ETV5 was found to be expressed exclusively in sertoli cells, but not in germ cells at various development stages in adult male mouse testis. Due to deletion of etv5 gene, adult male mice showed lack of spermatogonial stem cells as early as 5 weeks of age and gradually developed a sertoli cell-only syndrome [32]. A recent study also showed the expression of ETV5 protein in granulosa cells and etv5 mRNA in corpora lutea in adult mouse ovary [33, 34]. Subsequently, Eo et al. utilized the etv5^−/−^ female mouse to investigate the role of etv5 in the development of mouse embryo [35]. The authors showed that lack of etv5 gene remarkably arrested the embryonic development and inhibited the blastocyst formation, implying a potential role of ETV5 in embryonic development. Significantly, it has been confirmed a positive regulatory relationship between c-kit signaling and PEA3 transcription factors in gastrointestinal stromal tumor and colorectal mucinous adenocarcinoma [36, 37]. Therefore, we hypothesize that c-kit signaling affecting ETV5 expression may be also important for the development of pre-implantation embryos.

The aims of the present study were to use human tripronuclear (3PN) embryos resulting from one oocyte fertilized by two spermatozoa simultaneously during IVF and mouse 2PN embryos to investigate the potential role of c-kit signaling in human pre-implantation 3PN embryonic development.

In the present study, we first collect human 3PN embryos to investigate the potential role of c-kit signaling in human pre-implantation 3PN embryonic development. Moreover, mouse 2PN embryos are used to further confirm the molecular regulatory relationship between c-kit signaling and ETV5 in embryonic development.

## Methods

### Reagents

The following reagents were used: rabbit anti-c-kit (CST, USA), rabbit anti-phospho-c-kit (CST, USA), rabbit anti-Erk1/2 (CST, USA), rabbit anti-phospho-Erk1/2 (CST, USA), rat anti-c-kit (eBioscience, USA), mouse anti-ETV5 (Santa Cruz, USA), G-1™ (Vitrolife, Sweden), G-2™ (Vitrolife, Sweden), Human serum album (HSA) solution (Vitrolife, Sweden), Imatinib (Biovision, USA), and recombinant human SCF (rhSCF, R&D Systems, USA), U0126 (Biovision, USA).

### Patients

From Dec 2017 to Nov 2018, we enrolled 768 women (age: 28–32 years) who underwent IVF therapy in Reproductive Medicine Center of Jiangxi Provincial Maternal and Child Health Hospital in China. All patients were in good physical and mental condition. The study was approved by the Clinical Ethical Committee of Jiangxi Provincial Maternal and Child Health Hospital, and informed consents from patients were obtained before the initiation of the study. Patients with a history of the following procedures or disorders were excluded: ovarian surgery, radiotherapy or chemotherapy, premature ovarian failure, ovarian dysfunction, hyperprolactinemia, thyroid dysfunction, or PCOS.

### Controlled ovarian stimulation

Ovarian stimulation was performed with the use of a prolonged protocol. Briefly, standard full dose of gonadotropin-releasing hormone agonist (3.75 mg, GnRH-a, Ipsen, France) was used in the second day of menstrual cycle for down regulation. Pituitary down regulation (Endometrial thickness ≤ 5 mm, serum FSH < 5 mIU/mL, LH < 5 mIU/mL, E_2_ < 50 pg / mL) was confirmed with transvaginal ultrasound and endocrine examination after 30 days. Then, according to the patient’s age, body mass index, serum basal FSH levels, LH levels, estradiol levels and antral follicle count, initial doses of 75–112.5 IU/d of recombinant human FSH (Merck-Serono, German) were used. The time and dose of recombinant human FSH was adjusted according to ovarian response as monitored by serum estradiol levels and vaginal ultrasound. When the dominant follicle was≥19 mm in diameter or at least 2 follicles were ≥ 18 mm in diameter, recombinant human FSH was stopped and a single injection of 6000–8000 IU of hCG (Merck-Serono, German) was administered. Oocyte retrieval was performed 36–40 h later under transvaginal ultrasound guidance.

### Collection of human immature oocytes and 3PN zygotes

Human oocytes: human immature oocytes which were discarded during IVF program were collected. Subsequently, the oocytes were washed with PBS for three times (5 min each time). For immunofluorescence staining, the oocytes were immobilised on polyL-lysine processing slides, and kept at 37 °C for 10 min. After the oocytes were dried, they were fixed with 4% paraformaldehyde for 20 min and stored at − 80 °C.

Human 3PN embryos: under Chinese law, human embryos are not allowed to be created for research. Therefore, 3PN embryos resulting from one oocyte fertilized by two spermatozoa simultaneously were often used as an ideal model to study the human embryonic development before implantation [38–40]. In clinical, these embryos are normally discarded due to they may generate triploid pregnancies [41]. Evaluation of the number of pronuclei 16–20 h after insemination allows identification of such embryos, characterized by the presence of three pronuclei. 1200 3PN zygotes were collected and in vitro cultured in different experimental groups randomly. Subsequently, the embryos were washed with PBS for three times (5 min each time). The embryos were store at − 80 °C for future analysis. For immunofluorescence staining, the embryos were fixed with 4% paraformaldehyde and immobilised on polyL-lysine processing slides, and kept at 37 °C for 30 min. Then the embryos were dried and stored at − 80 °C.

### In vitro culture human 3PN embryos

Human 3PN zygotes were randomly distributed into three experimental groups based on the different cultured systems. SCF group: G-1™ (medium for culture of embryos from the pro-nucleate stage to day 3) or G-2™ (medium for culture of embryos from day3 to blastocyst stage) + HSA solution + rhSCF (100 ng/mL); SCF + imanitib (c-kit inhibitor) group: G-1™ or G-2™ + HSA solution + rhSCF (100 ng/mL) + imanitib (10 μM); Control group: G-1™ or G-2™ + HSA solution + PBS; All embryos of each group were cultured in a humid 5% CO_2_ atmosphere at 37 °C.

### Immunofluorescence staining of oocytes and embryos

Initially, oocytes or embryos were washed with PBS for three times (5 min each time) and then fixed with 4% paraformaldehyde for 30 min. Samples were rinsed with 1% Triton X-100 for 30 min; washed with PBS for three times (5 min each time). Non-specific binding sites were blocked with 5% goat serum albumin (Zhongshanjinqiao Biotechnology, China) for 60 min. Then, the samples were incubated with corresponding antibodies antibodies at 4 °C overnight, primary antibody was replaced with PBS in negative controls. The samples were taken out and kept at room temperature for 30 min, washed with PBS for three times (5 min each time), incubated with Cy3-conjugated corresponding secondary antibodies for 1 h at 25 °C in the dark, washed with PBS for three times (5 min each time). The samples were stained with DAPI (Zhongshanjinqiao Biotechnology, China) for identifying nuclei, and observed with a fluorescence microscope (Olympus, Japan).

### Animal experiment

All animals’ studies were carried out strictly under protocols approved by the Animal Care and Use Committee of Jiangxi Provincial Maternal and Child Health Hospital. 200 ICR (6-weeks) mice used for this study were housed in a temperature and humidity controlled room under a 12 h/12 h light/dark cycle. Female mice were superovulated by injection of 5 IU pregnant mare serum gonadotropin (PMSG; Sigma, USA), followed after 48 h by an injection of human chorionic gonadotropin (hCG; Intervet, Holland). Subsequently, the mice were mated overnight with males of the same strain. At 20 h after hCG injection, we collected one-cell embryos from the resected oviducts into modified human tubal fluid medium (HTF; Irvine Scientific, USA). The cumulus granulosa cells were removed by hyaluronidase (Sigma, USA). Next, mouse embryos were pooled and distributed into the three experimental groups, which were SCF group: G-1™ (medium for culture of embryos from the pro-nucleate stage to day 3) or G-2™ (medium for culture of embryos from day3 to blastocyst stage) + HSA solution + rhSCF (100 ng/mL); SCF + imanitib (c-kit inhibitor) group: G-1™ or G-2™ + HSA solution + rhSCF (100 ng/mL) + imanitib (10 μM); SCF + U0126 (MEK/ERK inhibitor) group: G-1™ or G-2™ + HSA solution + rhSCF (100 ng/mL) + U0126 (30 μM); Control group: G-1™ or G-2™ + HSA solution + PBS; All embryos of each group were cultured in a humid 5% CO_2_ atmosphere at 37 °C.

### Western blot

Total proteins were extracted from cells using the RIPA lysis buffer containing protease inhibitors (Applygen, China) and phosphatase inhibitors (Sigma, USA). The protein concentrations were determined by NanoDrop 2000c spectrophotometer using BCA protein assay kit (Applygen, China). After loading equal amount of protein samples, SDS-PAGE (12% sodium dodecyl sulfate polyacrylamide gel electrophoresis) was performed. The proteins were then transferred to a PVDF membrane (Merck-Millipore, USA). After blocking with Tris buffered saline containing 0.05% Tween-20 (TBST) and 5% non-fat dry milk or 5% BSA for 1 h, the membrane was incubated with corresponding antibodies at 4 °C overnight, washed in TBST, followed by incubation with the corresponding horseradish peroxidase-conjugated secondary antibodies for 1 h. Visualization of the proteins was detected with ECL chemiluminescence. Beta-actin (Santa Cruz, USA) was used as a loading control. The intensity values were assessed and analyzed with Image J software. (human 3PN embryos: *n* = 100 for per lane, mice 2PN embryos: *n* = 120 for per lane).

### RNA extraction and real-time PCR

Total RNA was extracted from embryos with RNeasy kit (Qiagen, China) according to the manufacturers’ instructions. Reverse transcription reactions were performed using Super cDNA First-Strand Synthesis Kit (CWBiotech, China), and the quantification of total RNA was performed on a NanoDrop spectrophotometer. Real-time PCR was performed in an ABI 7500 real-time PCR system (Applied Biosystems, USA) using Ultra SYBR Mixture with ROX (CWBiotech, China). The following primers were used: C-kit (Forward: AAGGACTTGAGGTTTATTCCT, Reverse: CTGACGTTCATAATTGAAGTC); ETV5 (Forward: AGGACCCCAGGCTGTACTTT, Reverse: TGGCCGATTCTTCTGGATAC); GAPDH (Forward: AGAAGGCTGGGGCTCATTTG, Reverse: AGGGGCCATCCACAGTCTTC). The reactions were incubated at 95 °C for 10 min, followed by 40 cycles at 95 °C for 15 s and at 60 °C for 1 min. All reverse transcription reactions included no-template controls; and all PCR reactions were run in triplicate. Relative gene expression was determined using comparative CT (2-ΔΔCt) method. (human 3PN embryos: *n* = 50, mice 2PN embryos: *n* = 90).

### Embryo assessment

A good quality embryo should consist of 7–9 blastomeres with a uniform size, and the fragment proportion should be less than 20% at day 3 for human 3PN embryos after fertilization (for mouse 2PN embryos was at day 2.5 after fertilization). The good quality embryo rate refers to the number of good quality embryos divided by the total number of all embryos. Blastocyst formation was determined and graded by using the system of Gardner and Schoolcraft [42]. The blastulation rate refers to the number of blastocysts divided by the total number of all embryos. The good quality blastulation rate refers to the number of good quality blastocyst divided by the total number of all blastocysts. Non-blastocysts are defined as embryos do not develop into blastocyst stage. The assessment was made in a blinded manner by two embryologists.

### Statistical analysis

The software package SPSS 17.0 (SPSS Inc., USA) was used for all data analysis. In general, results among experimental groups were analyzed by chi-square test. For all tests, *p*-value < 0.05 was considered statistically significant.

## Results

### C-kit was expressed in the human pre-implantation 3PN embryos

**Fig. 1:**
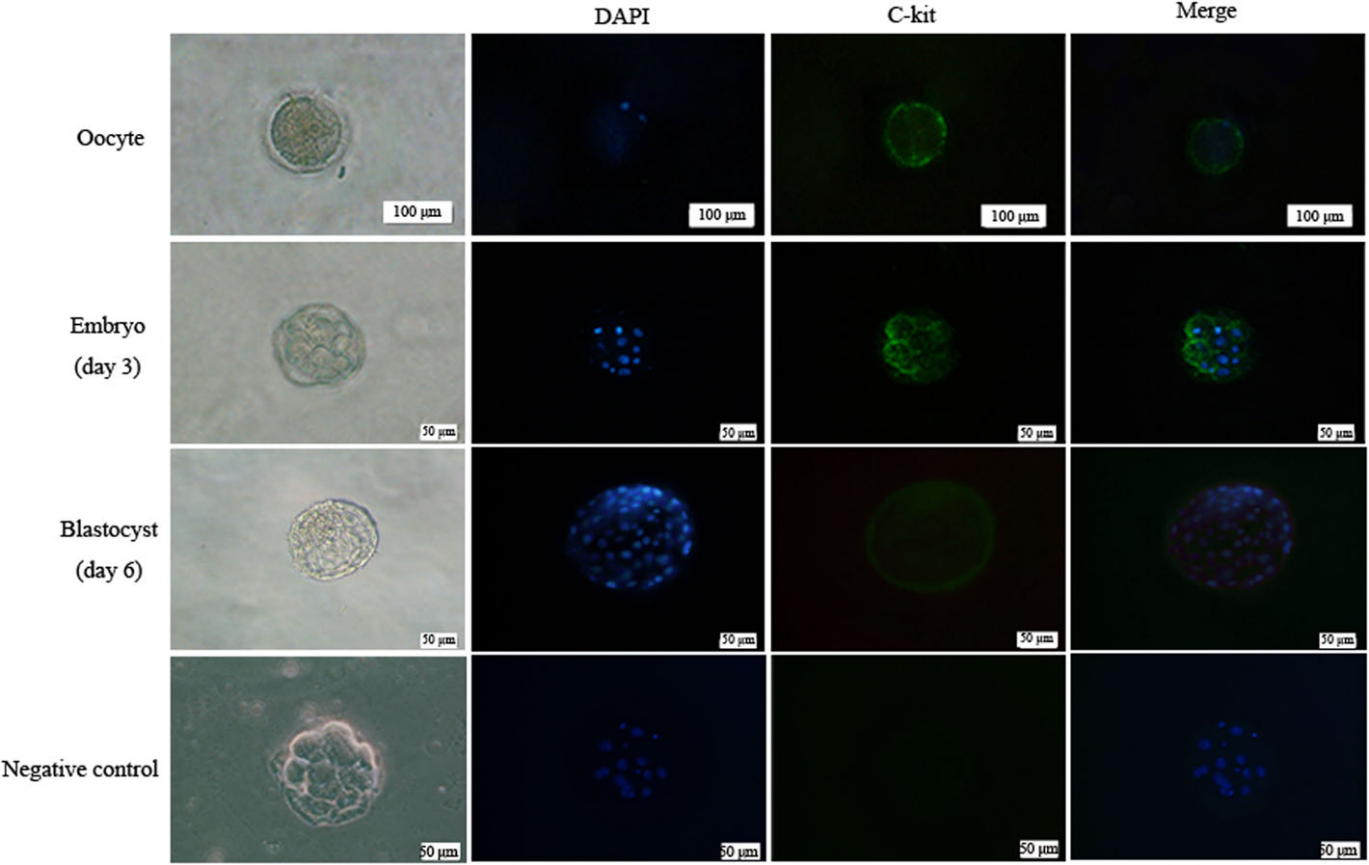
The expression of c-kit in human immature oocytes, 3PN embryos at eight-cell stage (day 3) and blastocysts developed from 3PN embryos (day 6). Immunofluorescence staining showed that c-kit protein was also expressed on the surface of human immature oocytes, the surface of blastomeres of human 3PN embryos (day 3) and the surface of human 3PN blastocysts (day 6). Negative control was stained with second antibody alone

Firstly, we investigated the expression of c-kit in human discarded immature oocytes, 3PN embryos at eight-cell stage (day 3) and blastocysts developed from 3PN embryos (day 6) by immunofluorescence staining, respectively. The results showed that c-kit protein was expressed on the surface of cells ubiquitously in all human immature oocytes, 3PN embryos and blastocysts developed from 3PN embryos (Fig. 1), implying that c-kit signaling might be closely correlated with human pre-implantation embryonic development.

**Table 1:** In vitro culture outcomes of human 3PN embryos in each group

	SCF	SCF + Imatinib	Control	*P-value*
No. of embryos	400	400	400	
Rate of good quality embryos	257/400 (64.25%)^a^	197/400 (49.25%)^b^	201/400 (50.25%)^b^	*< .01*
Rate of blastulation	234/400 (58.5%)^a^	182/400 (45.5%)^b^	198/400 (49.5%)^b^	*< .01*
Rate of good quality blastulation	189/400 (47.25%)	159/400 (39.75%)	165/400 (41.25%)	*> .05*

a vs b represent have statistic difference, b vs b represent no statistic difference

### Activation of c-kit signaling promoted human 3PN embryonic development and blastulation

Next, we in vitro co-cultured human 3PN zygotes with supplemental SCF, SCF + imatinib (c-kit inhibitor) and PBS (control group), respectively. Compared with SCF + imatinib group and control group, embryos co-cultured with SCF showed obviously higher rate of good quality at day 3 (SCF group: 64.25%, SCF + imatinib group: 49.25%, and control group: 50.25%, *P < 0.01*) and better rate of blastocyst formation at day 6 (SCF group:58.5%, SCF + imatinib group: 45.5%, and control group: 49.5%, *P < 0.01*). Although there was no statistic difference of good quality blastocyst formation between these groups, SCF group showed a higher rate than other two groups (SCF group: 47.25%, SCF + imatinib group:39.75%, and control group: 41.25%, *P > 0.05*) (Table 1). There was no statistic difference between SCF + imatinib group and PBS group. These data suggested that activation of c-kit signaling by SCF might promote human 3PN embryonic development and facilitate human 3PN embryonic blastulation.

### C-kit signaling increased ETV5 expression in human 3PN blastocysts

**Fig. 2:**
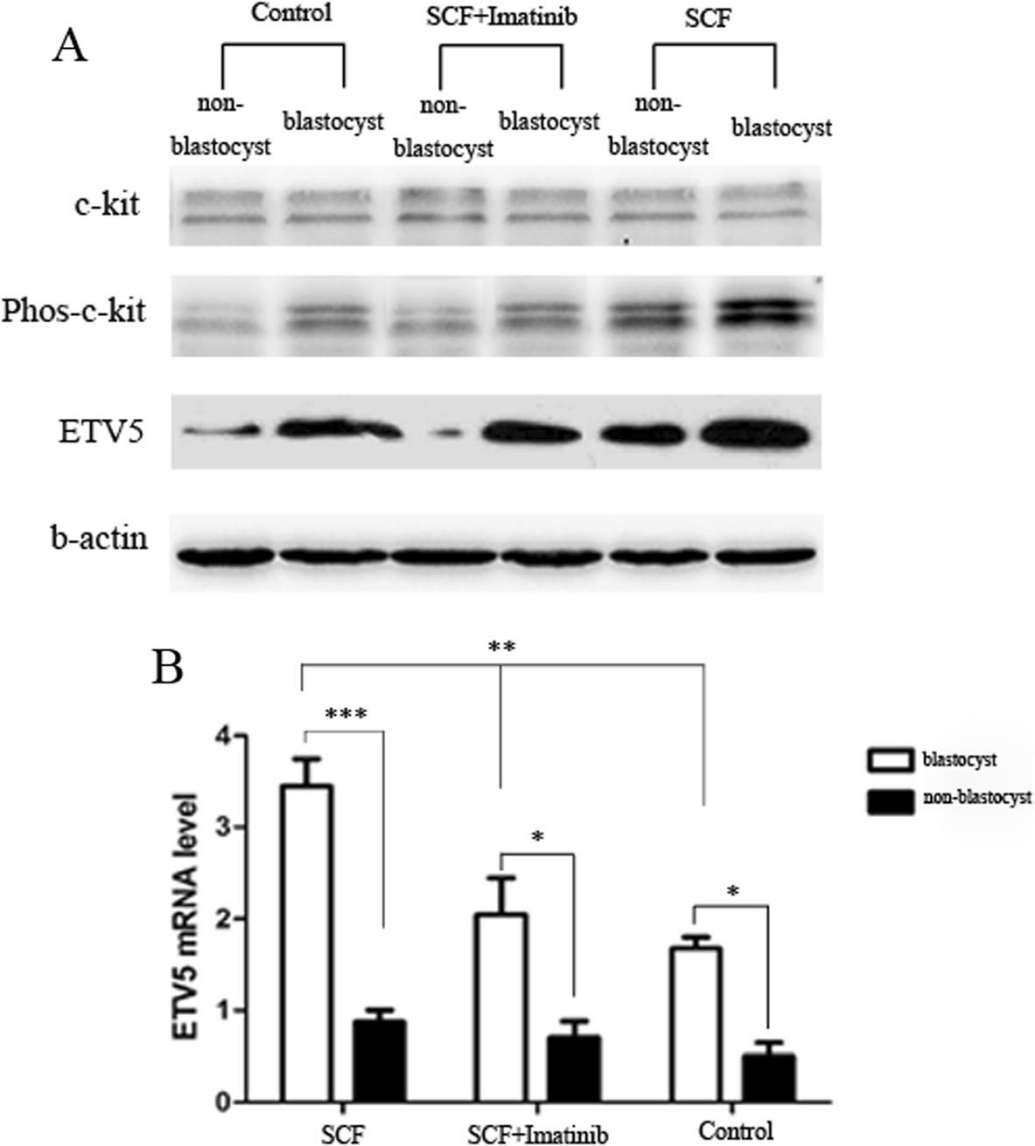
Comparison of the ETV5 expression in human 3PN embryos between SCF group, SCF + imatinib group and control group. Western Blot (**a**) and RT-PCR (**b**) showed that the expression of ETV5 was significantly higher in blastocysts than that in non-blastocysts in all groups. Furthermore, the results showed that the level of ETV5 was significantly higher in SCF group than that in other groups (*P < 0.05, ** *P < 0.01*, *** *P < 0.001*)

Then, we compared the expression of ETV5 between blastocysts and non-blastocysts (embryos were not developed into blastocyst) in these groups. As shown in Fig. 2, the expression of ETV5 mRNA and protein in blastocysts was higher in blastocysts than that in non-blastocysts in all groups. Interestingly, we also found that the level of ETV5 was significantly higher in SCF group than that in other groups (Fig. 2), indicating that the expression of ETV5 was likely to be regulated by c-kit signaling.

**Table 2:** In vitro culture outcomes of murine 2PN embryos in each group

	SCF	SCF + Imatinib	SCF + U0126	Control	*P*
No. of embryos	500	500	500	500	
Rate of good quality embryos	323/500 (64.6%)^a^	277/500 (55.4%)^b^	261/500 (52.2%)^b^	298/500 (59.6%)^b^	*< .05*
Rate of blastulation	292/500 (58.4%)	257/500 (51.4%)	266/500 (53.2%)	271/500 (54.2%)	*> .05*
Rate of good quality blastulation	257/500 (51.4%)^a^	211/500 (42.2%)^b^	201/500 (40.2%)^b^	231/500 (46.2%)^b^	*< .05*

a vs b represent have statistic difference, b vs b represent no statistic difference

### C-kit signaling promoted murine pre-implantation 2PN embryonic development and blastulation by up-regulating ETV5 expression via MEK/ERK pathway

**Fig. 3:**
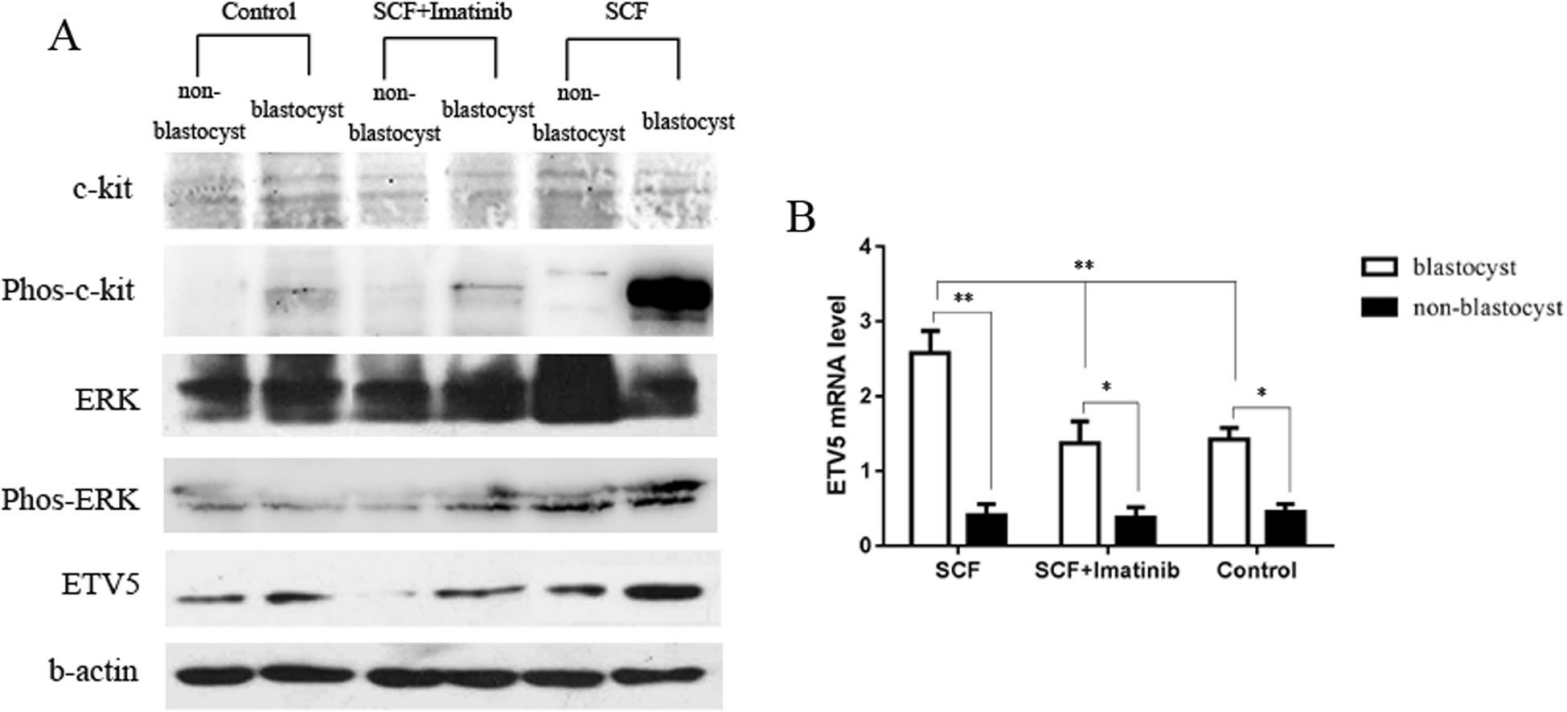
Comparison of the ETV5 expression and ERK phosphorylation in mouse 2PN embryos between SCF group, SCF + imatinib group and control group. Western Blot (**a**) showed an increased ETV5 expression and a higher phosphorylation level of MEK/ERK signal molecule in mouse embryos from SCF group than those from other groups. RT-PCR (**b**) showed that the expression of ETV5 was significantly higher in blastocysts than that in non-blastocysts in all groups (*P < 0.05, ** *P < 0.01*)

**Fig. 4:**
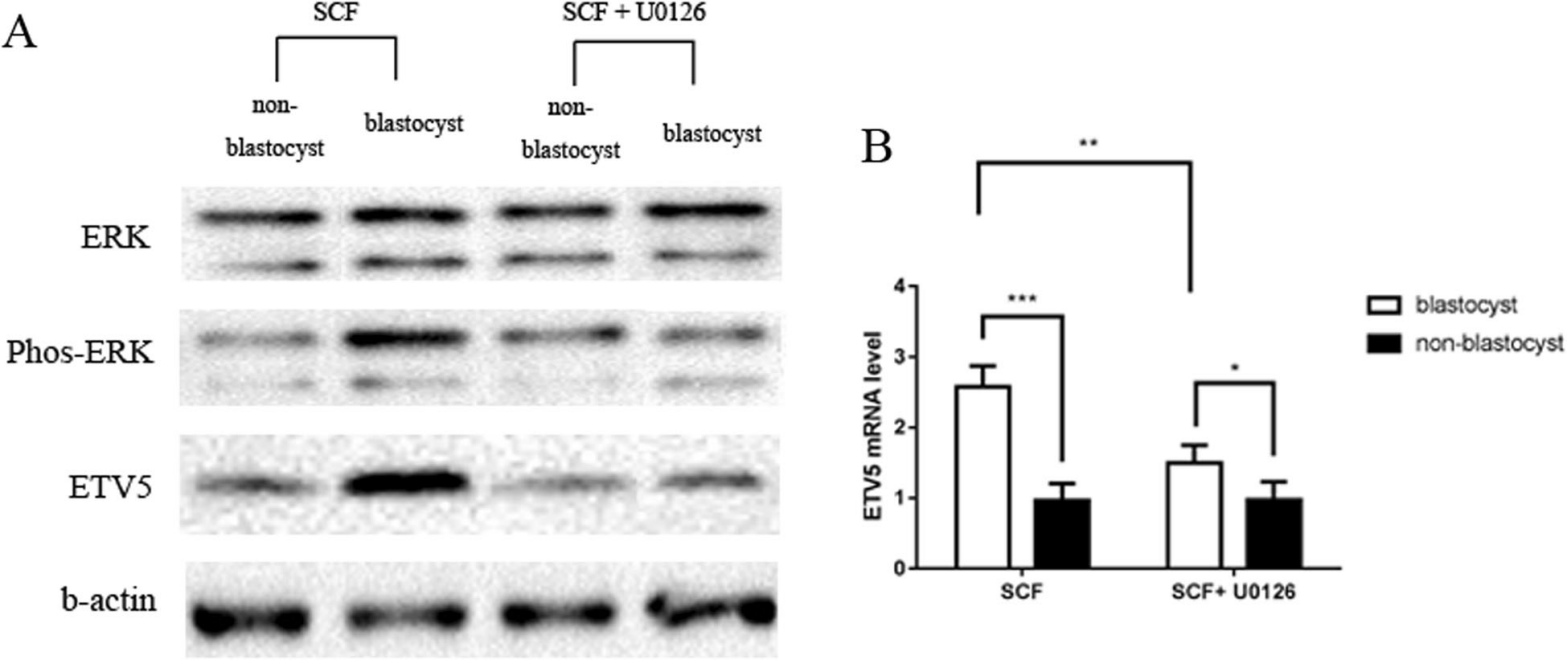
Comparison of the ETV5 expression and ERK phosphorylation in mouse 2PN embryos between SCF group, SCF + U0126 group. Western Blot (**a**) and RT-PCT (**b**) showed that after inhibiting MEK/ERK signaling activation by U0126, the expression of ETV5 was significantly decreased (*P < 0.05, ** *P < 0.01*, *** *P < 0.001*)

Subsequently, we next collected murine 2PN zygotes and co-cultured them with supplemental SCF, SCF + imatinib (c-kit inhibitor), SCF + U0126 (MEK/ERK inhibitor) and PBS (control group), respectively. Similar with the results in human 3PN embryos, embryos in SCF group showed higher rate of good quality (SCF group:64.6%, SCF + imatinib group: 55.4%, SCF + U0126 group: 52.2%, and control group: 59.6%, *P < 0.05*) and higher rate of good quality blastocyst formation (SCF group: 51.4%, SCF + imatinib group:42.2%, SCF + U0126 group: 40.2%, and control group: 46.2%, *P < 0.05*) than those in other groups (Table 2). Significantly, SCF group also showed a better rate of blastocyst formation, though there was no statistic difference (SCF group: 58.4%, SCF + imatinib group: 51.4%, SCF + U0126 group: 53.2%, and control group: 54.2%, *P > 0.05*). There was no difference between SCF + imatinib group, SCF + U0126 group and PBS group. Subsequently, western blot showed an increased ETV5 expression and a higher phosphorylation level of MEK/ERK signal molecule in embryos from SCF group than those from other groups (Fig. 3). Furthermore, inhibition of MEK/ERK signaling activation by U0126, the expression of ETV5 was significantly decreased (Fig. 4), and the promotion effect of embryonic development by SCF was also weakened (Table 2). All these results further implied that c-kit signaling might promote mouse pre-implantation embryonic development and blastulation by up-regulating ETV5 expression via MEK/ERK pathway.

## Discussion

Although advances in assisted reproductive technology (ART) have been rapid in recent years, the number of available embryos before implantation remains unsatisfactory. Under in vivo condition, oocyte is surrounded by granulosa cells, which provide nutrients to the oocyte. It is acknowledged that granulosa cells could secret multiple kinds of cytokines, which facilitated oocyte development, successful fertilization, embryogenesis and early stage embryonic development. However, granulosa cells are usually stripped, which is beneficial to observe zygote fertilization and embryonic development, in addition, the present in vitro culture system are still imperfect. Thus, thoroughly understanding the specific requirements for in vitro culturing early stage embryos are urgently needed.

SCF is one of the most important cytokines produced by granulosa cells. It can stimulate c-kit signaling and subsequently plays a key role during the primordial follicle activation and oocyte/follicular development [43–45]. Recent studies have detected the expression of c-kit gene and protein in murine embryos, and added exogenous SCF could promote the expansion of the surface area of the spreading blastocysts [46]. Taniguchi et al. reported that co-cultured murine embryos with granulosa cells increased the rate of embryonic development to late blastocyst and to hatching stage, which was probably related to the activation of c-kit signaling by SCF [26]. Additionally, Lim et al. in vitro cultured mouse embryos with exogenous SCF supplementation and showed that activation of c-kit signaling significantly supported early mouse embryonic development by accelerating the cleavage of blastomere, moreover, it was likely involved in stimulating AKT signal molecule [27]. These studies indicated c-kit signaling might play an important role in murine embryonic development.

Significantly, we showed here that c-kit was ubiquitously expressed from oocyte stage to blastocyst stage before implantation, implying a close relationship between c-kit and human pre-implantation 3PN embryonic development. To determine this relationship, we then in vitro co-cultured human 3PN zygotes with supplemental SCF, SCF + imatinib (c-kit inhibitor) and PBS, respectively. We found that addition of SCF significantly improved the rate of good quality embryo at day 3, the rate of blastocyst formation at day 6 and the rate of good quality blastocyst formation at day 6. These findings predicted that activation of c-kit signaling by SCF might promote human 3PN embryonic development, conversely, inactivation of c-kit signaling could weaken this promotion effects, which was observed in SCF + imatinib group and PBS group.

It has been documented that ETV5 was involved in the proliferation and differentiation in mouse embryonic stem cell by transcriptional regulating the expression of its target genes Gbx2 and Tcf15 [47]. Additionally, deletion or mutation of ETV5 gene would significantly result in inhibition of embryonic development and restriction of blastocyst formation in mouse [35, 48]. Accordingly, we speculate that ETV5 transcription factor may also play an important function in human embryonic development. Our results showed that the expression of ETV5 was significantly higher in blastocysts when compared with non-blastocysts, suggesting that ETV5 might be relevant for human blastocyst formation. The higher level of ETV5 in SCF group hints that activation of c-kit signaling by increased ETV5 in human 3PN embryo.

Although our results suggested a potential role of c-kit in human embryonic development and implied a possible positive regulatory relationship between c-kit and ETV5 transcription factor, all these findings were investigated in human 3PN embryos. As we known, 3PN embryo often shows aberrant chromosome numbers and is accompanied with inherently abnormal development process. For this reason, we subsequently collected mouse normal 2PN zygotes to further confirm our findings. As we supposed that add SCF improved the rate of good quality embryo, blastocyst formation, and good quality blastocyst formation, as well as the elevated ETV5 expression, supporting the results found in human 3PN embryos.

In fact, several studies have manifested the positive regulatory relationship between c-kit and PEA3 members (ETV1, ETV4, and ETV5) in multiple types of tumors. Chi et al. studied the role of c-kit signaling in gastrointestinal stromal tumors in mouse model and demonstrated that c-kit signaling enhanced the proliferation and invasion of tumor cells by up-regulating ETV1 via MEK/ERK pathway [36]. Furthermore, Tan et al. reported that c-kit signaling could promote the development of colorectal mucinous adenocarcinoma by increasing ETV4 expression through MEK/ERK pathway [37]. As mentioned before, ETV5 is the member of PEA3 subfamily, which possessed the similar structure and function as ETV1 and ETV4 [28]. Therefore, we deduce that maybe there is a similar regulatory mechanism between c-kit and ETV5 in embryo. In line with our hypothesis, a higher phosphorylation level of MEK/ERK signal molecule in SCF group signified that c-kit might stimulate MEK/ERK signal molecule to up-regulate the expression of ETV5 in embryo. Additionally, after inhibition of MEK/ERK signaling, the expression of ETV5 was significantly reduced and the rate of blastocyst formation was also decreased, further supporting our hypothesis.

In conclusion, our study revealed that c-kit signaling might promote the human pre-implantation embryonic development and blastocyst formation by up-regulating the expression of ETV5 via MEK/ERK pathway. Although the present study is based on the human 3PN embryos and mouse 2PN embryos, our results, at least in some ways, suggest a potential crucial role of c-kit in the development human pre-implantation embryo and provide new ideas in optimizing in vitro embryo culture condition during ART program. Further studies are necessary to confirm the function of c-kit signaling in human normal embryonic development, and thereby in order to establish a safe and effective in vitro embryo culture condition for infertile patients.

## Data Availability

All data generated and analyzed in this study are included in this published manuscript.
